# *Prima facie* reasons to question enclosed intellectual property regimes and favor open-source regimes for germplasm

**DOI:** 10.12688/f1000research.10497.1

**Published:** 2017-03-17

**Authors:** Madeleine-Thérèse Halpert, M. Jahi Chappell

**Affiliations:** 1 Falk School of Sustainability, Chatham University, Gibsonia, PA, 15232, USA; 2 Institute for Agriculture and Trade Policy, Minneapolis, MN, 55404, USA; 3 Centre for Agroecology, Water, and Resilience, Coventry University, Ryton-on-Dunsmore, Coventry, CV8 3LG, UK

**Keywords:** Agroecology, agrobiodiversity, germplasm, innovation systems, intellectual property, plant breeding, seed systems

## Abstract

In principle, intellectual property protections (IPPs) promote and protect important but costly investment in research and development. However, the empirical reality of IPPs has often gone without critical evaluation, and the potential of alternative approaches to lend equal or greater support for useful innovation is rarely considered. In this paper, we review the mounting evidence that the global intellectual property regime (IPR) for germplasm has been neither necessary nor sufficient to generate socially beneficial improvements in crop plants and maintain agrobiodiversity. Instead, based on our analysis, the dominant global IPR appears to have contributed to consolidation in the seed industry while failing to genuinely engage with the potential of alternatives to support social goods such as food security, adaptability, and resilience. The dominant IPR also constrains collaborative and cumulative plant breeding processes that are built upon the work of countless farmers past and present. Given the likely limits of current IPR, we propose that social goods in agriculture may be better supported by alternative approaches, warranting a rapid move away from the dominant single-dimensional focus on encouraging innovation through ensuring monopoly profits to IPP holders.

## Introduction

Given the challenges of sustainably providing food security for the present and future human population, it is often asserted that large-scale, technology-intensive agricultural innovation is necessary now, more than ever ( Beddington, 2010; Monsanto, 2015). Indeed, there seems to be near consensus, from corporations to social movements, that “business as usual is not an option” ( IAASTD, 2009; Joubert, 2016; Unilever, 2016). This sense of urgency is embraced by many agrifood corporations, who often put forward their products and services as key contributions to help society innovate into a better future. Leaving aside the flaws in this framing of the challenges facing us (see e.g., Lappé & Collins, 2015), large multi-national agricultural corporations are, in a certain sense, uniquely placed to lead in this innovation process: they increasingly dominate all aspects of the food system, including seeds ( Howard, 2016). Further, within the area of agricultural inputs, these corporations have been able to assure their continued prominence through the dominant intellectual property regime (IPR), particularly patents on seeds. Whether the approaches to IPR embraced by such actors is to the (public) good in the face of today’s large-scale problems is the topic of this paper.

The underlying claim made (especially, but not solely) by large agricultural corporations in support of intellectual property protections (IPP) is that “locking up” innovations behind patents is a necessary mechanism to ensure continued innovation. The *ex post* (“after-the-fact”) inefficiency that occurs when IPPs prevent other innovators from building on new technologies is widely recognized, but is considered part of a “profitable bargain for society” ( Moschini, 2010). The argument goes that in an area that involves high research costs, the net social good of innovations spurred by the potential monopoly protection of patents is greater than what is lost due to patents’ restrictions.

This logic is widespread and broadly accepted, and recent decades have seen an increase in the importance of IPPs in shaping seed systems around the world, particularly in the U.S. ( Kloppenburg, 2004; Luby & Goldman, 2016). Given the challenges of providing both food security and environmental sustainability in agriculture, agricultural corporations argue that innovation—and therefore patents—will only become more important yet.

 But what if patents pose more of an impediment than an aid to addressing current challenges? The actual balance of costs and benefits realized from intellectual property typically goes unquestioned. So it is possible, *prima facie*, that patents and similar elements of dominant global IP systems are unnecessary, and perhaps even inimical, to the development of socially-beneficial innovations in agriculture. Furthermore, alternative approaches may be equally or better able to support innovation through mechanisms that decrease or eliminate the *ex post* inefficiency ( Cimoli *et al.*, 2014). This paper explores these ideas, specifically with reference to germplasm, based on an analysis of existing and theoretical dynamics in agriculture and innovation.

## Intellectual property protections for plants in the U.S. and in international agreements

Since the 1960s, a jumble of international governing organizations have attempted to regulate IPPs for plant genetic resources. During this time, significant pressure by individual governments and international institutions has been exerted to adopt what could be called a “global IPR”, which has been “developed principally in the Western legal context… principally a U.S. utilitarian approach” ( Forsyth, 2016; see also Henry & Stiglitz, 2010).

The “utilitarian” innovation system of the U.S. is based on an approach whereby ideas and materials are putatively owned by individual entities, excluding all others from using such “intellectual property” without permission. Within this approach, three primary forms of IPP governing plants and plant genetic resources have been developed over the past century: plant patents; Plant Variety Protection certificates; and utility patents.

The 1930 Plant Patent Act allowed breeders to patent plant varieties that reproduce asexually (i.e., without seeds), protecting putative owners of IP while sidestepping controversies around seed saving practices ( Heald & Chapman, 2011). Starting from 1970, sexually reproduced plants were also “protected” through Plant Variety Protection (PVP), providing that the varieties could be determined to be novel, distinct, and uniform. PVP certificates included two important exceptions: a breeders’ exception allowing the use of protected varieties for non-commercial research and the development of varieties not essentially derived from the protected variety; and a farmers’ exception allowing seed saving for personal use ( Heald & Chapman, 2011; Pardey *et al.*, 2013). The third form of IPP in the U.S. has its origin in the 1980 U.S. Supreme Court case *Diamond vs. Chakrabarty*. This case (and subsequent rulings) asserted that utility patents were applicable to plant varieties, and even genetic sequences in certain cases ( Van Dooren, 2008). Unlike PVPs, the extension of utility patenting to plant and genetic materials involved no exceptions for seed saving, research, or other breeding activities. Certain forms of “dual protection” are also possible, combining different kinds of patents, or a PVP certificate and a utility patent ( Pardey *et al.*, 2013). Additionally, large commercial breeders have made use of contracts, laws protecting trade secrets, intra-industry-regulation and enforcement (“private ordering”) to exclude others from accessing their innovations ( Butruille *et al.*, 2015), and to reinforce formal modes of IPP. In some cases this has made IPP restrictions significantly more severe ( Elkin-Koren, 2005; Kloppenburg, 2014).

Many policies at the international level have paralleled the U.S.’s trajectory. For example, the International Union for the Protection of New Varieties of Plants (UPOV) establishes requirements that varieties to be protected are novel, distinct, uniform, and stable. While it had included farmers’ and breeders’ exceptions similar to that of PVPs, the most recent version of the agreement made these exceptions optional ( Salazar *et al.*, 2007; Van Dooren, 2008). Meanwhile, the World Trade Organization requires all member nations to have some form of IPP for plants through the Agreement on Trade-Related Aspects of Intellectual Property Rights (TRIPS). Under TRIPS, countries technically have the option to develop their own IPP systems, which could potentially support alternatives to the dominant global IPR ( Kloppenburg, 2010). However, in practice most countries simply adopt restrictions descending from the U.S./Western approach.

At times, however, international agreements have included approaches differing from this pattern. Some agreements have attempted to regulate plant genetic resources as common heritage, to address the uneven flow of germplasm from “developing” countries to “developed” ones, and to account for the crucial historical and continuing contributions of countless farmers to plant breeding ( Aoki, 2009). While these attempts are useful for suggesting alternatives to protecting and supporting innovation, they have so far had relatively little impact, and have been significantly constrained by the dominant global IPR. For example, the International Treaty on Plant Genetic Resources for Food and Agriculture (ITPGR) established a system in which recipients of germplasm from international seed banks cannot patent any of the seeds they receive from these banks ( Aoki, 2009). However, patenting germplasm or genetic materials that are subsequently *derived* from such multilateral system seeds or particular genes or DNA is allowed under the treaty ( *ibid*.). This means that there is a still the opportunity for patenting entities to benefit from common heritage while refusing to share subsequent benefits ( Vogel *et al.*, 2011).

## Draining the pool of knowledge: Enclosing more than giving back

While the necessity and effectiveness of IPPs, and patents in particular, are often assumed, some researchers have begun to examine the quality of these claims, especially in the context of germplasm. The pertinent question is essentially whether IPPs may limit future innovation more than they contribute to it. And patents, in particular, have been heavily scrutinized from this point of view. By granting exclusive rights of ownership to the patent holder, they are one of the most restrictive approaches to IPP, essentially allowing the owner to set the price for others to use their IP at infinity ( Stiglitz, 2014). Thus,

- “What seem to be more important [than strong IPRs] are the ‘opportunities,’ the potential for discoveries, related to the pool of knowledge to be exploited…
- …Patents inevitably enclose what would otherwise have been in the public domain. In doing so, not only do they impede the efficient use of knowledge, but because knowledge itself is the most important input into the production of further knowledge (innovations), they may even impede the flow of innovations,” ( Stiglitz, 2014).

Under Stiglitz’s models using “plausible conditions,” the incentives to innovation provided by patents encourages innovation initially, but ultimately does not sufficiently replace what it removes, stifling further innovation. This effect, combined with a possible underuse of existing innovations resulting from IPPs, is a dynamic that has been identified in biomedical research as the “anti-commons,” by Heller & Eisenberg (1998). In an anti-commons, “more intellectual property rights may lead paradoxically to fewer useful products for improving human health.” While Heller and Eisenberg clarify that they are not speaking about the “routine underuse” of innovations that occurs under patents, their description of an anti-commons appears to fit the results of Stiglitz’s model as well.

Given that agroecosystems span a wide range of cultural and environmental conditions and affect a wide variety of needs and impacts (from food security to their effects on wild biodiversity to climate change impacts), it would seem appropriate to have an innovation system that encourages greater accessibility to knowledge for a diversity of approaches and actors. Yet the current IPR appears to have contributed to the neglect of certain approaches to agriculture and plant breeding and contributed to a dearth of plant breeding PhDs ( Goodman, 2002; Vanloqueren & Baret, 2009). Further, Vanloqueren and Baret point out that practices like agroforestry that provide a number of public goods (e.g. sustainable livelihoods, resilience, and environmental quality) are based on system-level practices that are not patentable, and generate benefits over a long time period, two characteristics that strongly limit the pertinence of IPPs for boosting innovation. Such dynamics should be an object of concern; robust innovation systems ought to grant appropriate consideration to the full scope of ideas that could be socially useful ( van den Hove *et al.*, 2012).

To give a brief practical example of the dynamics at hand, one study of genetic diversity in pearl millet cultivars in India demonstrated that farmer social and management practices helped to maintain diversity and variation among local landraces, which “possess superior nutritional quality as well as higher fodder yield under severe conditions,” ( vom Brocke *et al.*, 2003). This is consistent with numerous studies showing that maintenance of agrobiodiversity can significantly contribute to sustainable farmer livelihoods, resilience, and adaptability ( Chappell *et al.*, 2013). But despite these useful properties, the diversity within landraces means they are generally not distinct, uniform, and stable, as would be required for protection under global IPR ( Salazar *et al.*, 2007). Further, the restrictions of typical IPPs means farmers would not be able to legally treat protected varieties “as raw material for direct use and further improvement [which] is still the norm in many parts of the world” ( *ibid*). Between this and the requirements of uniformity, distinctiveness, and stability, the dominant global IPR often exerts pressure to decrease diversity, and thus limit the usefulness and adaptability of our future seed supply—taking more out of the collective “pool of knowledge” than the IPPs put back.

 These dynamics represent the dangers of enclosing knowledge, but we have not yet covered the evidence as to how much IPPs do incentivize further innovation, that is to say, what IPPs give back to the pool of knowledge. On the one hand, drawing from case studies in the Pacific Islands region, Forsyth & Farran (2013) observed that “a Western IP system deflects attention from the need to support the organisations actually generating agricultural innovation in the region” (where breeding funding comes primarily from the public sector and NGOs who are not seeking patent-based returns on investment). Further, the dominant Western IPR may undermine traditions of benefit sharing and “undermine regional initiatives to promote food security through the sharing of plant genetic resources” ( Forsyth & Farran, 2013). In the cases they examined (and by analogy, they argue, many other “less developed countries”), prioritization of food security has generally been associated with supporting diversity, autonomy, and protection of farmers’ access to seeds within alternative and traditional networks, while approaches focusing on global IPR have often accompanied a trade-oriented mentality that does not truly address the needs and particularities of local communities ( Forsyth & Farran, 2013; see similar conclusions based on research in other “less developed countries” in Chappell *et al.*, 2013; McKeon, 2015).

However, in contrast to areas where the public sector dominates funding for plant breeding, IPPs should theoretically be responsible for significant outputs of research systems where plant breeding research is largely funded by private companies, as it is in the U.S. Heald & Chapman (2011) examined this hypothesis in one of the most extensive empirical analyses of IPPs to date. The authors studied the relationships between diversity, PVPs, patents, and commercially available varieties for apples and 42 vegetables over the period of 1903–2004. While a substantial number of new varieties were commercialized during this timeframe (which they took to represent innovation), only 3.8% of varieties commercially available in 2004 (excluding corn) were ever subject to patents, and only 16% of patented varieties were ever commercialized, suggesting a weak connection between IPPs and innovation in this area of breeding. In other words, most of the innovation in these plants was produced independently from IPP incentives. It should be noted that in the case of corn, patenting activity was much more prominent and patented seeds were prevalent in the market. But Heald and Chapman assessed that patents in corn may represent rent-seeking more than protection for innovation. That is, patents in corn may serve to exclude others from accessing “protected” germplasm without supporting any further innovation ( Heald & Chapman, 2011).

Even if this is the case, Western-style IPP might still be justified if rent-seeking owners use what they have withdrawn from the pool of knowledge to produce even higher-quality innovations than they would have otherwise produced. That is, the “monopoly rent” they extract from patents on, say, corn varieties may not spur them to innovate by creating *more* varieties, but it is possible that they use their profits to come up with *higher quality varieties*. However, in the case of plant breeding, there are reasons to doubt that extremely high-cost patented research is worthwhile in this manner, either. For example, research on seed prices has demonstrated that transgenic traits in commercialized seed are overpriced with regard to the relative research costs and yield gains provided to farmers through conventional breeding ( Goodman, 2002; Moss, 2013). In this case, IPPs may simply be enabling companies to charge prices for their IP that are actually greater than the benefits they produce ( Moss, 2013). A second, if tentative, line of evidence that patents may not be driving innovation of significantly superior varieties comes from a study by Bulte *et al.* (2014). Their analysis of randomized control and double-blind trials in Tanzania found that differences in yield between modern and traditional cowpea varieties were wholly due to differences in farmers’ management practices based on *perceived* differences in the varieties (as harvests were the same for farmers who received modern varieties and those who did know which type of seed they got). While this was only one study, and its results cannot be extrapolated to all modern seeds (or specifically those under IPPs), a comprehensive review by Loevinsohn *et al.* (2013) implies that the literature evaluating agricultural innovation is rife with similar challenges to scientific validity: they screened over 20,000 studies, and came up with only *5* that met reasonable standards of rigor (e.g., that would be capable of eliminating the confounding effects found by Bulte *et al.*).

In short, there is a significant lack of rigorous evidence that IPPs have led to the kind of higher-quality innovation that would justify their restrictions, much less sufficient evidence to establish that IPPs are decisively “giving back” more than they are taking from the collective pool of knowledge.

## Further considerations challenging contemporary dominant IPR

Beyond the dynamics related to the “pool of knowledge,” other factors may limit the appropriateness of global IPR for plant genetic resources: the connections between agriculture, plant breeding, and a number of other public goods. For example, with regards to “essential facilities”—resources that have no substitute and are fundamentally necessary for further innovation to occur— Henry & Stiglitz (2010) argue that no broad patents, and possibly, no patents at all should be granted. They give the specific example of genetically modified foods (e.g., crops), and cite Harhoff *et al.*’s (2001) conclusion that patents in this area may not only not be necessary to innovation, but may hold back socially useful applications. Even those who strongly defend the use of IPPs for seeds point out at the same time that society at large benefits from broad access to plant genetic resources and the ability to save seeds ( Scalise & Nugent, 1995). The problem with broad access, according to Scalise and Nugent, is that society’s benefits come at the cost of inventors, and therefore, society will see fewer important innovations by inventors and miss out on new technologies that will help feed everyone and improve general welfare. Beyond the challenges to this claim that we have already addressed, it is important to re-emphasize the large uncertainties present in evaluating the benefits of supposed innovations in plant breeding. That is: not only is the academic research on benefits of agricultural innovation lacking in rigorous and controlled studies, but as Stone *et al.* (2014) point out farmer decisions in many cases may be dominated by social learning (emulation) and lead to a high degree of “faddism”. In their study, farmer adoption of new varieties was dominated by cues taken from what other farmers were doing, and was not meaningfully related to the qualities of each set of new seeds. This was not due to some deficiency on farmers’ part; they note that “yields and profits from any given seed are highly variable”, and “attributing… performance advantages that have not been truly isolated from [their] confounding factors” is ubiquitous and long-standing throughout agriculture, echoing Loevinsohn *et al*.’s findings. The result is that it may, in practice, be impractical and unlikely that farmers will be able to decisively identify the benefits of innovations as quickly as private actors produce them, especially without the legal right to save and select seeds themselves, creating an information asymmetry benefitting IPP holders at the cost of farmers. One might term it as a problem of a “market for persimmons,” where farmers cannot quickly or easily distinguish the benefits of one seed versus another. (Various types of the persimmons fruit may appear similar, but need to be treated differently; cf. Akerlof (1970)).

Plant genetic resources are also tied to other public goods, such as biodiversity. For instance, agrobiodiversity affects the conservation of wild biodiversity ( Chappell *et al.*, 2013), and the loss of the former can negatively affect the latter. Therefore, if IPPs were leading to a loss of agrobiodiversity, that would be another argument against the dominant approach. Unfortunately, the degree of agrobiodiversity loss (or increase) over the past decades is difficult to measure and highly contended ( Montenegro de Wit, 2015). However, the loss of small independent seed companies is more straightforward to measure, and would imply some levels of lost diversity given that larger, consolidated seed firms will have higher incentives to breed for a small amount of “elite” lines with “‘broad adaptability’ – the capacity of a plant to produce a high average yield over a wide range of growing environments and years… [while] varieties yielding well in one zone but less in another are quickly eliminated” ( Desclaux *et al.*, 2012). With regards to seed company consolidation and IPPs, not only have the applications for plant and utility patents and PVPs risen steeply since the 1980s and 90s, but the percentage of patents and PVPs held by the top applicants has also increased ( Pardey *et al.*, 2013). Farmers have increased their reliance on purchased rather than saved seed over the same time period as consolidation has increased dramatically ( Howard, 2016; Marco & Rausser, 2008). In 2007, four companies (Monsanto, DuPont, Syngenta and Groupe Limagrain) controlled more than half of the global proprietary seed market ( ETC Group, 2008). And all of them except Limagrain are currently part of prospective mergers or buy-outs that would further increase consolidation throughout the agricultural input chain ( Purdy, 2016). Howard (2016) in fact states that “the seed industry is… nearing domination by just two firms”.

Many have expressed various concerns that such extreme concentration in this field has drawbacks for both food consumers and producers, depressing innovation and effectively allowing the industry to operate as an oligopolistic trust ( Howard, 2016; Moss, 2013; Moss & Taylor, 2014). Such a high level of concentration also creates a paradox of collaboration. That is, cross-licensing traits theoretically makes IPP less exclusive. However, since the small number of large seed companies often only cross license with each other, this form of “collaboration” may threaten potential for innovation by reducing competition within this group and reinforcing the concentration of power in the industry ( Howard, 2016; Moss, 2013). Indeed, in the case of germplasm, unlike other public goods, a depletion in current and future availability can be more related to a lack of use rather than overuse ( Montenegro de Wit, 2015). In this way, the feedback between industry consolidation and the use restrictions represented by IPPs in germplasm should be worrisome.

## Massively parallel computing? Peasant seed innovations vs. high technology centralization

As we have presented, contemporary global IPR has been tied to increased consolidation in germplasm research and commercialization, effectively centralizing a huge amount of resources for germplasm “innovation”. The centralizing tendencies of global IPR compared to various forms of decentralized breeding have different potential strengths. Centralization often offers greater precision in measuring results, but typically does so by eliminating environmental variance from its considerations, encouraging homogenization and limiting the actual applicability of its innovations to the large diversity of agricultural systems ( Desclaux *et al.*, 2012; Howard, 2016). In contrast, decentralized selection may allow greater farmer participation and allow breeding efforts to provide closer matches between innovations and diverse local conditions. Several lines of research have suggested that this may be the case ( Aistara, 2011; Desclaux *et al.*, 2012). Decentralized *in situ* breeding further potentially allows crops to be exposed to their wild relatives, and thereby may incorporate diverse genetic material into the breeding pool ( Jarvis & Hodgkin, 1999). While global IPR may in theory be compatible with this kind of *in situ* and decentralized efferts, in practice the very basis for these dynamics are restricted under dominant IPPs.

Another line of reasoning to prefer decentralized approaches reiterates Scott’s (1998) classic observations on how centralizing forces often necessarily seek to reduce or marginalize vital complexities that otherwise allow local communities to function, innovate, or thrive. The idea that improved innovation and problem-solving may come from allowing great access and freedom to use genetic resources for many decentralized actors is also analogous to the rationales and results from Massively Parallel Computing (e.g., Barney, 2016) and crowdsourcing (e.g., Brabham, 2008). Although these systems are not perfectly analogous, a baseline idea may still apply: that many different centers of experimentation and knowledge may solve problems more quickly than concentrating resources among a smaller number of specialists. And alternative approaches, like Participatory Plant Breeding, represent the potential to combine strengths of decentralization and expert knowledge ( Desclaux *et al.*, 2012).

Further, decentralized approaches may be better equipped to deal with the complex relationship between the diversity of cultural practices, crop biodiversity, and the diversity of localized and alternative IPPs ( Forsyth, 2016). Stability and distinctiveness requirements of current IPPs, as well as their emphasis on genetic factors above all else can pose obstacles to management strategies that produce valuable and adaptable, but “unstable”, cultivars. IPPs may also contribute to systematic failures in acknowledging, recognizing, and respecting the importance of cultural and social practices around seeds, including the meaningful patterns of association between cultural diversity and biodiversity generated by localized factors ( Desclaux *et al.*, 2012; Montenegro de Wit, 2015).

Practically speaking, decentralized selection can occur in both formal and informal settings. If done well, formal participatory approaches can balance centralized organization and decentralized selection, and can be more efficient (considering response to selection, adoption by farmers, and cost-benefit ratio) than conventional plant breeding programs ( Ceccarelli, 2015). Informal farmer breeding systems take a variety of forms, including highly collaborative community-scale efforts. For example, some informal community breeding projects have involved a small number of farmers interested in performing earlier stage breeding work, with many farmers later selecting from advanced lines and providing the land necessary to grow them ( Salazar *et al.*, 2007).

Bodies of theory on networks and innovation also suggest possible ** disadvantages to centralized systems (and thus, the current global IPR and concomitant increases in consolidation). Insofar as centralized systems represent increased connectivity between potential “nodes” of innovation or adaptation, they may resemble studies of connected populations and networks in economics and ecology. Findings in both of these fields reflect increased risks of systematic failures and large-scale collapse at high levels of connectivity ( Erola *et al.*, 2012; He & Deem, 2010; Noble *et al.*, 2015; Sensoy *et al.*, 2013). Further, the significant power inequalities represented by centralized, consolidated systems may be directly inimical to innovation, if recent research is to be believed ( Farrell & Shalizi, 2015; Page & Vandermeer, 2013).

The challenges and potential threats posed by the centralizing nature and unequal power relations present in many projects featuring public funding or international collaboration bear continued scrutiny. Minimizing or even eliminating the current global IPR may be necessary to allow for the most productive forms of decentralization, which might be better-suited to develop effective localized solutions in this realm. At the same time, even a radical revamping of global IPR may be insufficient to challenge centralizing and anti-egalitarian practices and structures, many of which operate on the assumption that centralization will result in desirable and worthwhile efficiency ( Brooks, 2011).

## Alternative innovation systems

Numerous proposals exist for alternative innovation systems for plant breeding and that may respond to some of the drawbacks of contemporary global IPR. Proposals range from those that modestly strengthen the public sector within the current regime to those that involve more fundamental dismantling of current IPPs for plant genetic resources. A few suggestions are outlined and discussed here.

Some authors have argued that IPPs, even with recommended improvements to their implementation, should be thought of as only one piece of a larger innovation system ( Henry & Stiglitz, 2010). However, one might step back even further and question, as others have done, the value of emphasizing the idea of “innovation” itself ( Russell & Vinsel, 2016; Van den Hove *et al.*, 2012). In considering innovation to be the ends, we risk overshadowing the importance of maintenance and building work that is not considered inventive, and we also might lose sight of the actual ends that the innovation process is meant to achieve. Thus, we may even consider innovation to be one part of a larger germplasm management system, rather than the point of it, and increase the recognition of the importance of conservation and seed saving activities that might not be considered “innovative”.

Even within the “innovation” frame, Henry & Stiglitz (2010) advocate strengthening mechanisms to reduce the *ex post* inefficiency created by the knowledge enclosures of dominant IPPs. For example, a liability approach, where anyone can access a given previous innovation for a fixed cost, and patents that are not “winner-take-all” could enable greater follow-on innovation. Incentivizing research with prizes for achieving specific goals, and increasing the amount of research provided through universities could similarly help keep outcomes of high-cost breeding research publicly available ( Henry & Stiglitz, 2010). These authors also explore the complex factors that have motivated people to contribute to highly successful open source software projects, suggesting that a similar structure could be effective in other areas, such as plant breeding.

However, given the properties of plant germplasm; the possible advantages of a decentralized approach; and that it can be considered a “commons” in many ways ( Luby & Goldman, 2016), one place to look for ideas on how to maintain or enhance it and its public use is the voluminous work on common property resource (CPR) management. The diversity of existing and traditional approaches to governing breeding and plant genetic resources in fact bears some similarity to the diversity of formal and informal approaches to governing CPRs, which were notably examined by Ostrom (1990). With reference to sustainably managing CPRs, Ostrom noted that “the centralizers and the privatizers” often advocated oversimplified solutions based on idealized notions of their own effectiveness. The models justifying their authority (e.g., the “tragedy of the commons”) often relied on what Ostrom called “extreme assumptions”, which could not be properly applied to smaller-scale CPRs. In contrast, as the official website of the Nobel Prize summarized the work for which she was awarded a Nobel, “in many, but not all, cases, allowing users to develop their own rules to regulate the use of common property results in the most efficient solution for managing those resources” ( Nobel Media AB, 2014). Whether this general finding applies specifically to the seed/germplasm commons is an open question. But its possible applicability does, *prima facie*, further imply a shifting of the burden of proof onto those claiming overall benefits from global IPR.

To this point, one specific alternative approach to the dominant IPR has been developing in the form of the Open Source Seed Initiative (OSSI). OSSI has attempted to create the foundation for a robust, protected commons for plant genetic resources. As OSSI has pursued an approach focused on personal commitments made by the communities breeding and using OSSI-pledged materials, it has embraced a “moral economy” as opposed to a formal and definitively legally enforceable regime ( CSA, 2014). OSSI is not currently attempting to coordinate with governments, and in this way the organization has perhaps had more freedom to directly contest the dominant IPR than those operating within that context. And while a “moral economy” approach may seem outdated from the perspective of the nearly-hegemonic Western-style global IPR, the literature on CPRs show that “informal” (i.e., non-state) institutions can be a powerful tool for managing a commons. Thus OSSI’s approach has come to focus on the “OSSI Pledge”: an agreement that can be printed on seed packets wherein by opening the packet, the opener agrees to not restrict others’ use of the germplasm or its derivatives, and to reprint the agreement on seed packets of all future derivatives of the seeds as well ( Open Source Seed Initiative, 2016). The protection of derivatives as unpatentable is a critical distinction between this approach and regulations, such as the ITPGR, that do not prevent such privatization and future enclosure of parts of the commons. The open source pledge also decentralizes the very act of participating in the protected commons (or opting out of intellectual property). Agreement and responsibility are relocated to the level of individuals transferring the seed packet and enforcement through community norms and building relationships of trust.

Kloppenburg (2010) and CSA (2014) discuss the potential for open-source approaches, like the OSSI pledge, to create more inclusive plant breeding communities, and also to democratize the use of the tools of plant breeding, such as genomic and transgenic techniques. At the same time, given the concerns about OSSI expressed by some farmers and researchers involved in the food sovereignty and agroecology movements, as well as Native American communities ( Breen, 2014), it is apparent that there is still work to be done to foster networks and relationships that fully serve all those who are managing biodiversity and agroecosystems, and that may truly serve as an alternative to global IPR.

It also may be possible for an open-source protected commons to coexist with IPP for germplasm, as some plant breeders participating in OSSI have considered releasing certain varieties through the open source system and protecting others under IP ( Miller, 2014). However, a thorough exploration of the implications of having a dual commons-IP system is necessary to determine if it would actually be able to provide the potential benefits of both types of systems. At first glance, it seems that doing so would effectively create two separate breeding pools, which may be undesirable. It also seems that it would be difficult to allow patenting traits or specific sequences when such material may also exist in protected-commons varieties, and there is the distinct possibility that breeders of certain high-quality materials may choose the dominant IPR over a protected commons. Thus, while it may be theoretically feasible for the two to co-exist, it may be that to be successful, a protected commons for plant genetic resources would eventually necessitate the end of the current system of IPPs. A significant possibility, and the one that we have tried to show that there is at least a *prima facie* argument for, is that this might not be a bad thing for innovation, or society in general.

## Conclusions

At this point, many questions remain around what a more effective germplasm management system might look like. Some researchers have identified the need for future research to apply scenarios and modeling methods to the study of seed networks ( Pautasso *et al.*, 2012). Perhaps these methods could also be used to project some of the outcomes of potential changes mentioned above, though as we have pointed out, at least some established economic models have already implied a more open-source/protected-commons IP system may actually *help* innovation.

That said, one lingering issue is whether any single IPR can effectively support innovations across all contexts, particularly with across so-called “developed” and “developing” countries ( Forsyth & Farran, 2013; Stiglitz, 2014). Similarly, there is room to debate how responsibilities for managing plant genetic resources might be organized with regard to various organizations and scales of government. As Merson (2000) has pointed out, tension may arise when efforts to offer protection for genetic resources in developing countries focus on implementing sovereignty over resources at the national level, while management of plant breeding and biodiversity may actually be occurring on smaller community scales.

Although the food sovereignty movement is concerned with many different issues within contemporary agrifood systems, certain challenges to seed sovereignty could potentially be made moot if a protected commons approach to plant genetic resources were to be the norm. Yet even then, questions would remain with regards to how entities at various scales might bear and exercise responsibilities to support plant breeding and conservation. Most likely, innovation systems that will be able to effectively support a more decentralized, diverse mix of approaches to developing localized plant varieties will need to be compatible with a wide range of governance structures as well. In other words, different geographies, ecosystems, and histories may mean that plant genetic resources are best managed by different sizes and types of organizations from one community to the next, and which acknowledge the many different types of existing intellectual property relations and (still-evolving) traditions ( Forsyth, 2016).

As global IPR is currently only becoming more entrenched, the most important step may not be to settle on which new approach might be best, but rather to call into question the appropriateness of a global IPR for plant genetic materials. Given the analysis we have presented, the possibility that the dominant system may well be failing to live up to its purpose, and indeed may be mitigating against its supposedly desired effect, must be seriously considered.

The failings of this system should not surprise us, although the vast majority of those working within the pressures of this regime are likely doing their best to develop socially useful plant varieties within current norms. However, the logic of global IPR assumes that the viability of plant breeding depends first and foremost on its ability to generate profit; such an attitude may be sadly unsurprising to those familiar with the steady history of sublimating food security to other goals ( McKeon, 2015). This observation in fact aligns with the pattern wherein societies that see plant breeding as a predominately economic and trade asset tend to implement IPPs, while those prioritizing breeding’s value to livelihoods and food security favor more open access systems ( Forsyth & Farran, 2013). Or as Henry & Stiglitz (2010) have said, “the presumption that profit-maximizing behavior is socially optimal is not always right” (p. 238). However, OSSI Executive Director Claire Luby has asked, “In a globalized system where multinational companies are the major drivers, do societies still get a choice?” If it were to be agreed that alternatives should be more seriously pursued, it would also be incumbent on those who truly wish to support socially-beneficial innovation to agitate within their societies and to their governments to make sure such choices are possible—particularly those of us living in geopolitically powerful countries (like the U.S.) who have strongly influenced global IPR.

In part, what’s needed now is to reassess what is seen as success in a management system for plant breeding. For example, we may instead choose to focus on its ability to support a wide range of actors and activities for stable management of biodiversity on many scales. We may consider that a working plant breeding system will necessarily facilitate widespread access, exchange, and use of seeds, support decentralized efforts for local adaptation, and justly recognize the work of farmer-breeders today and over millennia. Therefore we must begin instead from these goals, and then ask what regulations or structures might encourage the dedication of the resources that will be necessary to support them, rather than assuming the status quo is the best system. Although any large-scale transition towards alternatives poses serious challenges, this is true of any attempt to fulfil the grand challenges of sustainable, food secure, and resilient agrifood systems. As many in the movements for food sovereignty and agroecology have proposed, working to improve participation, autonomy, political agency, and especially power imbalances will be indispensable in *any* attempt to prioritize these values and foster a more just and sustainable world ( Chappell *et al.*, 2013; Farrell & Shalizi, 2015; Perfecto *et al.*, 2009).
