# Supplementary material for: Prima facie reasons to question enclosed intellectual property regimes and favor open-source regimes for germplasm
Source: F1000Res. 2017 Mar 17;6:284. [Version 1] doi: 10.12688/f1000research.10497.1 (PMC5414820; doi:10.12688/f1000research.10497.1)
Supplement: Peer review history

# Peer review: Referee response for version 1

**DOI:** 10.5256/f1000research.11312.r21085

**Published:** 2017-07-12

**Authors:** Guntra A. Aistara, Ana Ruth Martinez Rizo

**Affiliations:** 1 Department of Environmental Sciences and Policy, Central European University, Budapest, Hungary

In this opinion essay, the co-authors Halpert and Chappell question whether conventional intellectual property protections (IPPs) for germplasm contribute to or limit socially beneficial innovation in the face of today’s large-scale agricultural challenges of simultaneously achieving food security and environmental sustainability. They present a clear argument, supporting their opinion with relevant literature.

 The essay is excellent and the conclusions are balanced and justified based on the evidence and arguments presented. Nevertheless, the topics covered raise a few further observations on the history of these debates that continue to create obstacles for alternative IP regimes, the exploration of which would help contextualize the current paper and hoped for solutions. These revolve around the definition of the “common heritage of mankind;” the separation of breeders from farmers; and the breeders’ and farmers’ exceptions present in UPOV-related regimes as opposed to patents. These debates have undergirded discussions about access to and sharing of benefits from agrobiodiversity, and may continue to pose obstacles for the types of alternatives suggested by the authors, necessary for fostering a more “just and sustainable world” (p. 9).

 The authors summarize the US 1930 Plant Patent Act (PPA), the Plant Variety Protection (PVP) certificates in force since the 1970s, and the 1980 *Diamond vs. Chakrabarty *US Supreme Court case, which allowed utility patents to be used for plant varieties. These IPPs may also be combined in “dual protection” systems, or reinforced and spread through international treaties such as the Union for Plant Variety Protection (UPOV) and the World Trade Organization Trade Related Intellectual Property Rights (TRIPS) agreement. One exception to more restrictive IPPs is the FAO International Treaty on Plant Genetic Resources (ITPGR), which limits the opportunity to obtain patents on genetic materials received from international gene banks, though it does not prevent the patenting of genetic materials subsequently derived from those materials.

 Halpert and Chappell review critiques and evidence that IPPs may be taking more from the knowledge pool than they are giving back, in effect creating an “anti-commons” (citing Heller & Eisenberg 1998). They argue that IPPs should encourage further research to create more diversity, and cite studies showing that sometimes the opposite is true, therefore indicating a weak, or even inverse relationship between IPPs and innovation in plant breeding. The authors refer to the inability of farmers to quickly distinguish the benefits of one seed versus another, and emphasize how difficult it is to evaluate the benefits of the supposed innovation in plant breeding, a fact that in the end can benefit IPP holders at the expense of farmers. The authors then explain how the fact that a few companies now control the global seed industry negatively affects genetic diversity and is facilitated by the IPPs.

 Finally, Halpert and Chappell analyze the centralizing tendencies of IPPs and compare them to decentralized breeding schemes, showing evidence of the benefits of decentralized in situ management strategies. The authors introduce alternative innovation systems, such as the Open Source Seed Initiative (OSSI). OSSI functions through a “pledge” printed on seed packets, whereby users agree not to restrict anyone else’s use of the germplasm or future derivatives of it. While this alternative of creating a “protected commons” offers individuals a way to opt out of dominant intellectual property regimes, the authors submit that an exploration of the implications of the coexistence of open source and dominant IPPs will be necessary in order to evaluate the long-term effects on plant breeding and access to seeds.

 The authors conclude that even if there are still a lot of questions to be answered about alternative open source/protected commons intellectual property regimes, it is necessary to question the assumption that dominant IP regimes are the only option that encourage innovation, and may in fact deter it, proposing that we may need to rethink what we mean by success in plant breeding.

***Common heritage versus common concern***

 The authors frame their inquiry in terms of “socially beneficial innovations,” but this raises a question about who defines the public good and how? The authors note that “some agreements have attempted to regulate plant genetic resources as common heritage” (p. 4), citing the ITPGR as an example. It is important to note, however, that even the ITPGR is not framed as “common heritage”, and it was exactly debates over whether or not plant genetic resources are a part of the “common heritage” of humankind that began the slow slide from a global commons approach to the dominant IPP approach. The concept of “common heritage” does not have one agreed definition, but was nevertheless diluted to an unspecified “heritage of mankind” in the ITPGR and a “common concern of humankind” in the CBD, because there has been no agreement on this issue since negotiations began on the FAO International Undertaking on Plant Genetic Resources in the 1980s (Brush 2007 ^1^, Murillo 2008 ^2^). This history of disagreement will also make a ‘return’ to a protected commons approach more difficult.

 This is also significant because if the heritage of genetic resources is not seen as “common,” it is necessary to separate out groups who are the true “heirs” of these resources, and thus  considered more suited to act on behalf of the “common concern of humankind” than others. These groups will thus be granted privileged access to the resources, or rights to further develop them. Although the ITPGR “ recognizes the enormous contribution farmers have made to the ongoing development of the world’s wealth of plant genetic resources,” the farmers’ rights protections in the treaty have never had the same legal standing as IPPs, which enforce breeders’ rights to develop and protect varieties. This has created a separation of farmers from breeders, and established breeders in effect as the rightful heirs and developers of genetic resources.

***Farmers versus breeders***

 The authors rightly point out that due to the broad range of cultural and environmental factors that affect plant breeding and growing practices, “it would seem appropriate to have an innovation system that encourages greater accessibility to knowledge for a diversity of approaches and actors” (p. 4). This would presume, however, that all of these different knowledge systems are equally valued, which has not been the case at least since the beginning of the dominant IP regime development, which has consciously enforced a separation between farmers and breeders and their knowledge systems.

 All new plant varieties protected under IPPs have been created using genetic materials that are a co-product of natural selection and farmer selection. Van Dooren (2008) ^3^ provides an insightful analysis of how foregrounding the “invention” of seeds or varieties by breeders, over the intricate webs of farmer and non-human interactions that have come before them, and on which they depend, results in fetishism of the seed. He observes that this creates a division between breeders as true “inventors” who are representative of “culture,” versus farmers who remain trapped as a part of “nature.” This also prioritizes the investments of individuals or institutions in the present and future over historical ones (Aistara 2012 ^4^).

 This separation of farmers from breeders forces a separation of scientific knowledge from traditional knowledge and creates scientific experts who are seen as better able to manage important global concerns. The UPOV system was originally differentiated from patents, in that discoveries of natural genetic mutation or cross- pollination, often made by farmers, could also be protected, which was not possible under patent law. Subsequently the UPOV treaty was also changed to require “discovery and development” rather than just discovery (UPOV 2002 ^5^), narrowing the interpretation of breeding to fit laboratory settings more readily than farmers (Aistara 2012 ^4^). This separation becomes especially important once different “exceptions” are applied to the different groups.

***Exceptions versus exclusions***

 As the authors note, “Unlike PVPs, the extension of utility patenting to plant and genetic materials involved no exceptions for seed saving, research, or other breeding activities” (p. 3). Indeed one of the main differences between patents and the UPOV regime and PVPs is that UPOV includes both a breeders’ exception and a farmers’ exception. There are fundamental inequalities between these exceptions, however. While the breeders’ exception allows breeders public access to protected germplasm for research purposes and the development of new varieties, the farmers’ exception, since the 1991 revision of UPOV, allows farmers to replant varieties for consumption purposes only. With the increasing narrowing of the farmers’ exception, similarities between UPOV and patents increase. Even in cases where the farmers’ exception does apply, it is sometimes so narrowly economically defined that it excludes income-generating farmers (Aistara 2012 ^4^). More importantly, the farmers’ exception does not allow a space for farmer breeding using protected varieties. What is specifically allowed by exception for some (breeders) becomes a criminal act for others (farmers) (ibid).

 The different functions of the exceptions are indicative of how dominant IPPs define innovation. The breeders’ exception is meant to allow for innovation, while the farmers’ exception is only meant to allow for consumption. If farmers are seen as consumers, rather than also potential breeders, they are effectively excluded from the possibility of innovation, despite the fact that is was farmer innovation over generations that created the diversity of varieties upon which breeders now depend. By separating the act of farming from breeding, it also separates the intangible versus tangible aspects of knowledge that surround farmer breeding, and that have been passed down with seeds over generations (Aistara forthcoming ^6^). Losing this knowledge by constricting the space for farmer breeding practices may hinder future innovation.

 The future-oriented idea of innovation also excludes many of the reasons why farmers’ may have innovated, and thus bred diversity, in the first place. There are benefits and uses from farmer-bred varieties that reach much beyond yield, and these other values and uses of seeds are also excluded from innovation in the future, because farmers do still tend to cross their own varieties with protected varieties, as noted by the authors (citing Salazar 2007). The farmers’ exception thus functions more as exclusion. Furthermore, farmer breeding is tightly interwoven with farmer seed exchange practices and social networks, which may have been what gave the diversity of seeds meaning in the first place, and may thus be the inspiration for innovation (Aistara 2011 ^7^). Cutting social networks of exchange through the imposition of IPPs (Strathern 1996 ^8^, Aistara forthcoming ^6^) may thus also cut off future innovations.

 While patents have been mainly used in the US, UPOV has dominated in Europe and is spreading throughout many developing countries as a requirement of free trade agreements. There has recently been a push to move towards plant patents for conventional breeding techniques in Europe as well, which would eliminate the breeders’ exception. Ironically, the European Seed Association, a breeders’ trade association, opposed patents for conventional breeding techniques or based on “purely biological processes” (as opposed to biotechnology) for precisely the same reason that farmer-breeders have opposed their own exclusion from the breeders’ exception:

 “ The breeders’ exemption is the cornerstone of a system that successfully balances the protection of individual intellectual property with the common interest of society to introduce innovation broadly and quickly by allowing free access for further research and breeding. This decision has the potential to not only restrict this free access to quite a number of products, but also to generally discourage breeding efforts in areas covered by such patents in the future.”

 The ESA 2012 Position on Intellectual Property Protection for Plant-Related Inventions in Europe even goes on to claim that the breeders’ exception “ can be regarded as a kind of ‘open source’ system and has always been relied upon by breeders for further improvement on each other’s varieties and boosted innovation in plant breeding.” This reinforces the fact that the question is really about who is included or excluded from the definition of breeder and thus has the exclusive rights to derive benefits from improving plant genetic resources that have already been improved upon for over 10,000 years. Dominant IPPs still try to create “privately public access” for some to derive exclusive benefits from such germplasm while excluding others (Aistara 2012 ^4^), while true open source systems like the OSSI try to both broaden that space of access, and prevent certain groups from deriving unfair benefits at the expense of others from open source germplasm in the future.

***Open Futures?***

 As the authors indicate, many questions remain unresolved both about how open source or other alternative innovation systems might work in practice, and how they will co-exist with dominant IPPs. For example, rather than simply “building relationships of trust” (p. 8), how well would the OSSI pledge, still a hybrid between formal and informal systems, work in systems where farmers may lack skills to read and write or be reluctant to enter even into semi-formal agreements? How would the OSSI pledge work for acknowledging innovation at the community rather than individual level, for example in areas such as the Potato Park in Peru, and how does it intersect with current legal norms (Martinez Rizo, forthcoming ^9^)?

 Halpert and Chappell suggest that rather than starting from the existing system, we should start from a set of goals and structure the system to accommodate it. Indeed in the meantime the first step would be to reform dominant IP regimes, including exceptions in both patents and UPOV/PVP systems. But rather than maintaining unequal exceptions for particular types of actors, exceptions should be made available for particular types of activities or purposes, such as for the maintenance of diversity, for research and development, for food security purposes, or even for small-scale business development purposes. This would already be a step towards maintaining a more open playing field and ending the segregation of actors and knowledge systems. Each such purpose will require different norms than the current requirements of novelty, distinctness, uniformity and stability, as well as options for recognizing community as well as individual contributions to breeding. OSSI and other open source seed initiatives would then be well placed to further broaden the spectrum of alternative approaches to facilitate both diversity and justice in seed politics.

We have read this submission. We believe that we have an appropriate level of expertise to confirm that it is of an acceptable scientific standard.

## References

[1] BrushS.B.: The demise of ‘common heritage’ and protection for traditional agricultural knowledge. *Biodiversity and the law: intellectual property, biotechnology and traditional knowledge*.2007;297-315

[2] MurilloJ: Common concern of humankind and its implications in international environmental law. *Macquarie Journal of International and Comparative Environmental Law*.2008;5:113-147

[3] van DoorenT: Inventing Seed: The Nature(s) of Intellectual Property in Plants. *Environment and Planning D: Society and Space*.2008;26(4) : 10.1068/dtvd 676-697 10.1068/dtvd

[4] AistaraG: Privately public seeds: competing visions of property, personhood, and democracy in Costa Rica’s entry into CAFTA and the Union for Plant Variety Protection (UPOV). *Journal of Political Ecology*.2012;19:127-144

[5] UPOV: The notion of breeder and common knowledge in the plant variety protection system based upon the UPOV convention Geneva.2002;8

[6] AistaraG: Organic Sovereignties: Struggles over Farming in an Age of Free Trade: In Press. *University of Washington Press*.

[7] AistaraG: Seeds of kin, kin of seeds: The commodification of organic seeds and social relations in Costa Rica and Latvia. *Ethnography*.2011;12(4) : 10.1177/1466138111400721 490-517 10.1177/1466138111400721

[8] StrathernM: Cutting the network. *Journal of the Royal Anthropological Institute*.1996;517-535

[9] Martinez RizoA: The implementation of an open source seed approach in Parque de la Papa, Peru: Innovation and knowledge from a community perspective: In Press. *Central European University*.

# Peer review: Referee response for version 1

**DOI:** 10.5256/f1000research.11312.r22291

**Published:** 2017-04-27

**Authors:** Sue Farran

**Affiliations:** 1 Northumbria Law School, Northumbria University , Newcastle, UK

This is an interesting article which makes a number of robust criticisms of the application of IPR to germplasm and linked to this consequences of food security. It is very wide ranging and treads a sometimes difficult and not always successful path between the global and the local. Indeed while the current global IPR regime is clearly unsatisfactory, and this is a case well made, it is not always clear if the solution also lies at a global or more nuanced local and diffuse level.

 For clarity given the potential international readership it should be made clear what aspects and information are USA located and what not.

 More use might have been made of literature and examples from Africa and India in respect of food security, IPR and seeds/germplasm. Nevertheless, this article makes an important contribution to the debate on the stranglehold of the current global regime and usefully identifies a number of areas needing further research.

 A further strength of the article, which is introduced but not yet fully explored, is drawing from other disciplines and theoretical models to challenge the claimed benefits of the current regime.

I have read this submission. I believe that I have an appropriate level of expertise to confirm that it is of an acceptable scientific standard.

# Peer review: Referee response for version 1

**DOI:** 10.5256/f1000research.11312.r21368

**Published:** 2017-04-19

**Authors:** William F. Tracy

**Affiliations:** 1 Department of Agronomy, University of Wisconsin–Madison, Madison, WI, USA

This is an opinion article on issues surrounding intellectual property regimes. It is well written and easy to read. As an opinion piece it cites previous publications and does not generate new data or knowledge. The main thesis of the article is that intellectual property regimes in cultivated plants are "neither necessary nor sufficient to generate socially beneficial improvements in crop plants and maintain agrobiodiversity". The authors go further and dispute the benefits of IP in spurring innovation and greater gains in plant improvement, a conclusion many would dispute. I am quite open to this argument and the authors do a good job citing literature to support their position. The authors also discuss the negative effects of IP on 'collaborative and cumulative plant breeding', a conclusion with which, I think, most observers would agree. They also blame the use of IP for the decline of pubic plant breeding at universities, NGOs, and International Centers. While IP may have a role in this decline, neoliberal economic policies have been the main culprit. In fact we can locate the rise of  IPP in plant breeding on those economic policies. Despite this quibble over the decline in public plant breeding, I tend to agree with, or at least am open to, many of the authors arguments.  

 My main concern with the article is that while they did an extensive literature review of papers that support their point of view, the review of the extensive body of opposing literature is almost non-existent. The few papers they mentioned are often from companies, which some readers might dismiss out of hand. Many readers, especially those antagonistic to the authors' thesis, will dismiss the current article because it does not address any of the numerous opposing publications and the data they include. I suggest the authors start by reviewing Smith et al., 2016 ^1^ and the literature contained therein. This is a review that comes to a diametrically opposed conclusion from the current paper. It has an extensive citation list supporting their conclusions. I will state here that I do not agree with many of the conclusions and extrapolations in Smith et al. ^1^ but it and many other articles opposing the conclusions of the current piece are out there and they need to addressed be the authors for this article to be taken seriously.

 Finally, in the authors' promotion of open source proposals, they recognize the concerns of food and seed sovereignty movements, but they do not address the chilling effect of the derivative clause on the widespread use of germplasm developed with public funds. To justify public support of breeding programs the germplasm needs to be made available as widely as possible with freedom to operate. Many public and private breeders will not use open source material because of restrictions on ownership of derivatives.

I have read this submission. I believe that I have an appropriate level of expertise to confirm that it is of an acceptable scientific standard, however I have significant reservations, as outlined above.

## References

[1] SmithSLenceSHayesDAlstonJCoronaE: Elements of Intellectual Property Protection in Plant Breeding and Biotechnology: Interactions and Outcomes. *Crop Science*.2016;56(4) : 10.2135/cropsci2015.10.0608 10.2135/cropsci2015.10.0608

# Peer review: Referee response for version 1

**DOI:** 10.5256/f1000research.11312.r21078

**Published:** 2017-04-10

**Authors:** Sheryl D. Breen

**Affiliations:** 1 Political Science Environmental Studies, University of Minnesota Morris, Morris, MN, USA

Halpert and Chappell provide a knowledgeable, well-written overview of global intellectual property protections and plant genetic resources and present a set of arguments for moving toward an alternative open-source approach. As they point out in their introduction, the widespread assumption is that monopoly patents on germplasm can pass a cost-benefit analysis test by creating paths for future innovation. The authors question the truth of this assumption, however, and ask whether alternative approaches such as the open-source framework also can support agricultural innovation while decreasing the *ex post *inefficiency of the intellectual property regime.

**Intellectual property protections for plants in the U.S. and in international agreements:** This section describes the evolution of a global intellectual property regime, primarily following the utilitarian approach of the United States and its development of plant patents, Plant Variety Protection (PVP) certificates, and utility patents. The authors provide a brief, useful summary of each stage in this development within U.S. law as well as the parallel international moves toward the International Union for the Protection of New Varieties of Plants (UPOV) and the World Trade Organization’s requirements in the Agreement on Trade-Related Aspects of Intellectual Property Rights (TRIPS). While alternatives have been possible, the authors write, they have remained limited within the global intellectual property rights regime.

**Draining the pool of knowledge: Enclosing more than giving back:** The authors ask whether intellectual property protections in fact erect barriers against future innovation, contrary to the widespread assumption, and thus decrease rather than augment our collective knowledge of agriculture and plant breeding. Using both theoretical arguments and empirical examples, Halpert and Chappell describe the way that plant patents promote the “anti-commons,” suppressing potentially valuable avenues of research. They present evidence that raises significant doubts about the validity of claims that plant patents lead to more or better commercial varieties and conclude that intellectual property protections for germplasm have not demonstrated their ability to promote needed innovation, much less net contributions to collective knowledge.

**Further considerations challenging contemporary dominant IPR: **This section provides further evidence that the global intellectual property regime may harm public goods, including biodiversity. The authors point out that research on agricultural innovation so far has been unsatisfying and that farmers’ decision-making on varieties does not provide reliable evidence of seed performance. In contrast, the seed industry has rapidly consolidated and is dominated by a small number of corporations.

**Massively parallel computing? Peasant seed innovations vs. high technology centralization:** The authors compare the costs and benefits of centralized systems for plant breeding innovation in the global intellectual property regime and decentralized selection as practiced by peasant farmers. Unlike the centralized system, peasant farmers’ *in situ *practices promote integration with local soil and climate conditions and allow exposure to wild relatives and resulting increases in biodiversity. Drawing from theories of Massively Parallel Computing and crowdsourcing, the authors suggest that decentralized problem-solving is more efficient and can take both formal and informal approaches. In contract, the centralized intellectual property regime is less able to recognize and respond to localized cultural and social practices, is prone to power differences that stifle innovation, and may be at higher risk of large-scale failure.

**Alternative innovation systems: **So far, the authors have challenged the belief that the global intellectual property regime is necessary and efficient in terms of plant breeding innovation. In this section, they lay out possible alternative systems, including revisions within the plant patent system, the use of common property resource management, a protected commons envisioned by the Open Source Seed Initiative, the establishment of a dual commons-intellectual property system, and – most radically – the elimination of intellectual property protections for plant genetic resources. These alternatives are not explored at length in this section, but the authors present a significant portfolio of possibilities that, in various ways, question the foundational assumptions beneath the intellectual property system and call for innovation in new directions.

**Conclusions:** The final section brings the authors to two concluding questions: 1) Can a single, centralized system of germplasm management encompass the wide range of traditions, practices, and relationships surrounding plant genetic resources? 2) How do we define “success” in a plant breeding management system? In other words, what are our goals? The authors suggest that multiple approaches are more promising than a unified global system and that the goal of profit maximization is limited and, when monopolistic, harmful.

 Overall, this article follows well-recognized standards of argumentation and includes credible, authoritative sources in its review of the literature. Two minor corrections are necessary, both in the conclusion:

1. Page 8, left column, second and third line of final paragraph: either “with” or “across” needs deletion;
2. Page 8, right column, third paragraph: Quotation from Claire Luby is not cited.

I have read this submission. I believe that I have an appropriate level of expertise to confirm that it is of an acceptable scientific standard.
